# Utilization of Fertility Treatment in Japan During the First Year of Insurance Coverage: Analysis of Real‐World Health Claims Data

**DOI:** 10.1002/rmb2.70033

**Published:** 2026-03-06

**Authors:** Makoto Okawara, Tomoichiro Kuwazuru, Moe Masunaga, Kenji Fujimoto, Masako Nagata, Takeshi Iwasa, Yoshihisa Fujino

**Affiliations:** ^1^ Department of Preventive Medicine and Community Health, School of Medicine University of Occupational and Environmental Health, Japan Fukuoka Japan; ^2^ Department of Environmental Epidemiology, Institute of Industrial Ecological Sciences University of Occupational and Environmental Health, Japan Fukuoka Japan; ^3^ Data Science Center for Occupational Health University of Occupational and Environmental Health, Japan Fukuoka Japan; ^4^ Department of Occupational Medicine, School of Medicine University of Occupational and Environmental Health, Japan Fukuoka Japan; ^5^ Department of Obstetrics and Gynecology, Institute of Biomedical Sciences Tokushima University Graduate School Tokushima Japan

**Keywords:** fertility clinics, health, infertility, insurance, medical records, reproductive techniques

## Abstract

**Purpose:**

In April 2022, fertility treatments, including assisted reproductive technology (ART), became eligible for public health insurance coverage in Japan. This study describes the prevalence and characteristics of infertility diagnoses and fertility treatments during the first year of coverage.

**Methods:**

This study analyzed claims data from 14 health insurance associations from April 2022 to March 2023. Infertility diagnoses and fertility treatments, and related comorbidities were identified via diagnosis, procedure, and drug codes.

**Results:**

Among 590,006 women and 619,551 men, the highest prevalence of infertility was seen in women (3.9%) and men (0.5%) aged 30–34 years. General fertility treatment was more common among younger women but decreased with age, while ART became more prevalent after age 35. The major prescriptions were hormone preparations for ART and luteal insufficiency among women. The diagnosis ratio of OHSS was higher than previously reported, with 8.0% to 9.6% of women undergoing fertility treatment, particularly receiving ART and younger. Male prevalence of diagnosis and treatment remained low, with multiple factors underlying access to treatment.

**Conclusions:**

Public insurance coverage improved access to fertility treatment and enhanced data standardization in Japan. This study indicates the need for targeted strategies to broaden equity in reproductive health services.

## 1 Introduction

The ongoing decline in Japan's birth rate and aging of the population are reaching critical levels. The 2023 vital statistics recorded approximately 730,000 births, and the total fertility rate fell to a record low of 1.20 [1]. By 2025, the first members of the baby boomer generation will be aged 75 or older, and the more than 20 million people falling into this age group represent approximately one in six of the total population. The proportion of people aged 65 and over will reach 30% of the total population, resulting in a situation where two working‐age individuals support one elderly person. These demographic changes pose numerous challenges for Japan, including increased social security expenses, a shortage of workers, and a declining total population.

Since the first reported birth resulting from in vitro fertilization and embryo transfer in 1983, fertility treatments including assisted reproductive technology (ART) have advanced in Japan [2]. Following confirmation of safety and efficacy through extensive research and clinical practice, births resulting from ART have increased annually. By 2021, approximately 600 facilities performed ART treatments, resulting in the birth of 70,000 newborns through ART [3]. Based on this background, some fertility treatments including ART became covered by health insurance in Japan in 2022. This reduced the individual financial burden per cycle to approximately JPY10,000 for artificial insemination and around JPY200,000 for in vitro fertilization or intracytoplasmic sperm injection. An upper limit on personal expenses based on income was also implemented. Under insurance coverage, restrictions on ART treatment are imposed based on the female patient's age. If the woman is under 40 years old at the start of treatment, the maximum number of cycles is limited to 6 per child. For women aged 40 to under 43, the maximum is 3 cycles. ART is not covered by insurance for women aged 43 and older.

The implementation of public health insurance coverage for fertility treatments represents a watershed moment in Japanese reproductive healthcare, fundamentally altering accessibility to ART and general fertility services across the nation. This historic policy shift, enacted in April 2022, transformed fertility treatments from privately funded or subsidy‐dependent services to comprehensively insured medical procedures, potentially affecting millions of couples experiencing infertility. The critical issue concerns how this unprecedented expansion of coverage translated into actual utilization patterns during its inaugural year of implementation. Understanding these utilization patterns is essential not only for evaluating policy effectiveness but also for identifying gaps in service delivery and planning future reproductive health strategies. Japan's demographic context, with its total fertility rate at a record low of 1.20 and approximately 40% of couples concerned about infertility, makes this analysis particularly urgent [1, 4]. Moreover, with one in 10 births now resulting from ART procedures, the patterns of fertility treatment utilization directly impact national demographic trajectories [5].

Prior to insurance coverage, fertility treatment access in Japan was constrained by substantial financial barriers, with some couples spending millions of yen on ART procedures, creating significant disparities in access based on socioeconomic status [6]. The transition to insurance coverage theoretically democratized access, but the actual patterns of utilization—who sought treatment, what treatments were pursued, and how gender differences manifested—remained unknown. Furthermore, while insurance coverage standardized billing through medical fee receipts, creating an unprecedented opportunity for population‐level analysis, no comprehensive examination of these newly available data had been conducted. The availability of detailed claims data from multiple health insurance associations offered, for the first time, the possibility to reveal actual treatment patterns beyond the limitations of institutional surveys or patient self‐reports. The Japanese Ministry of Health, Labour and Welfare released the National Database of Health Insurance Claims as open data, enabling certain analyses [7, 8]. However, these data represented the total number of claims; critical individual‐level aspects remained uncharacterized: the true prevalence of infertility diagnoses across different demographic groups, the differences in treatment utilization between men and women, and the types of fertility treatments most commonly pursued under the new coverage system. Thus, despite the substantial policy change and its potential demographic implications, the actual landscape of fertility treatment utilization in Japan during this first year remained largely uncharacterized.

Here, we analyzed comprehensive health insurance claims data from multiple insurance associations to elucidate the prevalence and characteristics of infertility diagnoses and fertility treatment utilization during the first year of public insurance coverage in Japan. This population‐level investigation sought to provide the first comprehensive understanding of how Japan's historic shift to insurance coverage affected real‐world fertility treatment utilization patterns and to identify potential disparities in access and treatment between different population groups.

## 2 Methods

This study was conducted under an observational design using medical claims data provided to the Data Science Center for Occupational Health at the University of Occupational and Environmental Health, Japan. We used medical receipts and insured person registers provided by 14 health insurance associations, with anonymization of each insured person. The observation period was from April 2022, when fertility treatment was first covered by public health insurance, until March 2023. The study included 590,006 female and 619,551 male insured persons aged 18–69 years and was approved by the Ethics Committee of the University of Occupational and Environmental Health, Japan (approval number ID24‐016).

Three definitions related to infertility and fertility treatment in individuals were created for this study. First, individuals diagnosed with infertility defined using the registered diagnosis. Second, patients who received infertility‐specific treatments. We used treatment codes for general fertility treatment management fees, ART management fees, oocyte retrieval, testicular sperm extraction (TESE), and more. These codes were based on fertility treatments that were newly covered by insurance in 2022. This definition represents the most conservative estimate of the proportion of individuals receiving fertility treatment, as inferred from claims data, serving as a definition unambiguously specific to fertility treatment. And third, recipients of a fertility treatment who were diagnosed with infertility and received fertility treatment, such as a clomiphene citrate or letrozole prescription. This patient group, when counted together with the second group of patients who received infertility‐specific treatments, represents a broader treatment utilization including patients receiving fertility treatment at medical institutions unable to bill for management fees. Table S1 shows the definitions of infertility diagnoses and fertility treatments for men and women. Infertility diagnoses and fertility treatment were defined using medical diagnoses and practices based on discussions within the study group, which included medical doctors with experience in infertility treatment and ART. We used ICD‐10 codes, injury and disease codes, medical procedure codes, drug codes and drug effect classifications to identify specific diseases and medical procedures.

Next, to confirm the validity of the defined disease names, we conducted a preliminary check of coverage among patients who had received infertility‐specific treatments. Tables S2 and S3 show the coverage ratios based on the defined disease names and representative disease names observed but not included in the definitions. We verified the disease names of all cases not covered by the defined disease names, and examined whether any coded disease names should be added to the definitions. The most common diagnoses given to patients not captured by the definitions were ovarian dysfunction, ovulation disorder and luteal dysfunction in women; and varicocele, hypogonadism and sperm abnormalities in men. Following discussions within the research group, we decided that these diagnoses would not be included in the definition of infertility diagnosis in this study. This is because if these diagnoses were included, individuals prescribed hormone replacement therapy for menopause or low‐dose estrogen‐progestin control pills (combined oral contraceptive) for dysmenorrhea or amenorrhea, and individuals who underwent varicocele surgery or treatment for hypogonadism (e.g., Kallmann syndrome), which are not directly related to fertility treatment, would also be included in the infertility diagnosis. Next, all cases with unencoded free‐text diagnoses were reviewed, and those considered to be related to infertility or fertility treatment (e.g., “infertility requiring ART” or “ovulation induction for unexplained infertility”) were recorded as unencoded infertility diagnoses.

In addition, to describe the diagnoses of diseases that may arise in connection with fertility treatment among patients undergoing fertility treatment, the following diseases were identified using ICD‐10 codes and disease names: ovarian hyperstimulation syndrome (OHSS); bleeding in the ovaries or abdominal cavity; salpingitis; pelvic inflammatory disease (PID) after oocyte retrieval; PID after artificial insemination; PID potentially associated with oocyte retrieval or artificial insemination; torsion of the ovary, ovarian pedicle and fallopian tube; and embolism and thrombosis of vein.

Finally, we described data on the number of patients diagnosed with infertility, those receiving fertility treatment, and those receiving infertility‐specific treatments. We also conducted overviews of diagnoses among patients receiving fertility treatment; the type of treatment and medications among patients undergoing infertility‐specific treatments; and diagnoses of diseases that may arise in connection with fertility treatment among patients undergoing fertility treatment and in women who were not diagnosed with infertility or received fertility treatment, categorized by age group.

As a preliminary analysis, we examined in detail the utilization of artificial insemination and oocyte retrieval, the diagnostic status of OHSS by treatment type, and the implementation status of AMH measurement. Furthermore, among patients who underwent oocyte retrieval, we assessed the association between age and OHSS using logistic regression, with OHSS as the dependent variable and age as a continuous predictor specified by a restricted cubic spline with five knots. Age‐specific predicted probabilities of OHSS and their 95% confidence intervals were derived from the model using predicted margins and presented graphically. All analyses were performed using Stata (Stata Statistical Software release 18.0; StataCorp LLC, TX).

## 3 Results

Tables 1 and 2 show the total number and percentage of men and women diagnosed with infertility and receiving fertility treatment or infertility‐specific treatments by age group. The highest percentages in each category were in the 30–34 age group, with 5.0% of women and 1.9% of men meeting the definition of an infertility diagnosis and 3.9% of women and 0.5% of men meeting the definition of fertility treatment. The highest percentage of recipients of infertility‐specific treatments was 2.6% for women and 0.3% for men in the 30–34 age group.

**TABLE 1:** Percentage of women receiving fertility treatment by age group.

Age group	Enrolled women (*N* = 590,006)								
	Number enrolled	Infertility patients							
		Total (*n* = 6853)		Individuals diagnosed with infertility^a^ (*n* = 6832)		Fertility treatment recipients^b^ (*n* = 5167)		Infertility‐specific treatments recipients^c^ (*n* = 3392)	
	*N*	*n*	%	*n*	%	*n*	%	*n*	%
18–24	71,501	91	0.13	89	0.12	71	0.10	15	0.02
25–29	38,817	877	2.26	874	2.25	651	1.68	386	0.99
30–34	42,351	2135	5.04	2129	5.03	1630	3.85	1091	2.58
35–39	52,352	2236	4.27	2229	4.26	1721	3.29	1203	2.30
40–44	62,001	1242	2.00	1239	2.00	970	1.56	659	1.06
45–49	78,270	227	0.29	227	0.29	116	0.15	37	0.05
50–54	88,637	38	0.04	38	0.04	7	0.01	1	0.00
55–69	156,077	7	0.00	7	0.00	1	0.00	0	0.00

^a^ Those registered with a defined infertility diagnosis between 1 April 2022 and 31 March 2023.

^b^ Those who were diagnosed with infertility and received defined treatments or prescriptions, in addition to infertility‐specific treatment recipients.

^c^ Those billed for general fertility treatment management fees, assisted reproductive medical care management fees, and fertility treatment procedures covered by public health insurance in 2022.

**TABLE 2:** Percentage of men receiving fertility treatment by age group.

Age group	Enrolled men (*N* = 6,19,551)								
	Number enrolled	Infertility patients							
		Total (*n* = 3033)		Individuals diagnosed with infertility^a^ (*n* = 3030)		Fertility treatment recipients^b^ (*n* = 850)		Infertility‐specific treatments recipients^c^ (*n* = 519)	
	*N*	*n*	%	*n*	%	*n*	%	*n*	%
18–24	83,948	27	0.03	27	0.03	5	0.01	2	0.00
25–29	53,694	359	0.67	359	0.67	83	0.15	45	0.08
30–34	53,262	990	1.86	988	1.85	262	0.49	158	0.30
35–39	59,740	869	1.45	868	1.45	270	0.45	180	0.30
40–44	67,013	503	0.75	503	0.75	150	0.22	90	0.13
45–49	76,059	192	0.25	192	0.25	62	0.08	35	0.05
50–54	79,221	65	0.08	65	0.08	15	0.02	8	0.01
55–69	146,614	28	0.02	28	0.02	3	0.00	1	0.00

^a^ Those registered with a defined infertility diagnosis between 1 April 2022 and 31 March 2023.

^b^ Those who were diagnosed with infertility and received defined treatments or prescriptions, in addition to infertility‐specific treatments recipients.

^c^ Those billed for general fertility treatment management fees, assisted reproductive medical care management fees, and fertility treatment procedures covered by public health insurance in 2022.

Tables S2 and S3 show coverage ratios by named disease category. Among those who received infertility‐specific treatments, 99.29% of women and 99.23% of men were captured by the defined disease names. Inclusion of unclassified disease names increased the capture rate to 99.38% for women and 99.42% for men. Among those who received clomiphene prescriptions, a treatment specifically designed for fertility issues, 93.26% of women and 94.74% of men were captured. Sensitivity analysis incorporating varicocele into the diagnostic definition captured 99.42% of infertility‐specific treatments; adding varicocele to the definition of infertility did not contribute to a substantial increase in capture rate.

Tables 3 and 4 show infertility diagnoses in patients receiving fertility treatment. The most common diagnosis among women was female infertility, followed by infertility, functional infertility, secondary infertility and primary infertility. The most common diagnosis among men was male infertility, followed by oligospermia, infertility and functional infertility.

**TABLE 3:** Disease name registration based on defined disease names among women.

	Fertility treatment recipients (*n* = 5167)		Ages 18–24 (*n* = 71)		Ages 25–29 (*n* = 651)		Ages 30–34 (*n* = 1630)		Ages 35–39 (*n* = 1721)		Ages 40–44 (*n* = 970)		Ages 45–49 (*n* = 116)		Ages 50–69 (*n* = 8)	
	*n*	%	*n*	%	*n*	%	*n*	%	*n*	%	*n*	%	*n*	%	*n*	%
Defined disease names																
Female infertility	2161	42	28	39	276	42	676	41	722	42	413	43	45	39	1	13
Infertility	1988	38	27	38	264	41	630	39	661	38	350	36	49	42	7	88
Functional infertility	682	13	3	4	62	10	190	12	258	15	158	16	10	9	1	13
Secondary infertility	547	11	4	6	42	6	150	9	207	12	133	14	11	9	0	0
Primary female infertility	495	10	6	8	91	14	174	11	140	8	72	7	12	10	0	0
Functional female infertility	433	8	0	0	44	7	113	7	166	10	100	10	10	9	0	0
Primary infertility	316	6	7	10	54	8	131	8	71	4	47	5	6	5	0	0
Fallopian tube obstruction	158	3	0	0	18	3	65	4	47	3	26	3	2	2	0	0
Fallopian tube stenosis	148	3	0	0	29	4	47	3	48	3	22	2	2	2	0	0
Female infertility of tubal origin	115	2	1	1	17	3	36	2	32	2	26	3	3	3	0	0
Pituitary infertility	58	1	1	1	7	1	15	1	28	2	6	1	1	1	0	0
Female infertility of cervical origin	35	1	0	0	4	1	18	1	9	1	4	0	0	0	0	0
Cervical mucus secretion insufficiency	29	1	0	0	2	0	11	1	10	1	6	1	0	0	0	0
Infertility of ovarian origin	20	0	1	1	2	0	6	0	6	0	5	1	0	0	0	0
Female infertility of uterine origin	3	0	0	0	1	0	1	0	1	0	0	0	0	0	0	0
Unencoded infertility diagnoses^a^	124	2	1	1	15	2	42	3	45	3	20	2	1	1	0	0

^a^ General fertility treatment, infertility requiring assisted reproductive technology, luteal insufficiency, ovulation induction for infertility caused by pituitary dysfunction, functional female infertility (male factor), etc.

**TABLE 4:** Disease name registration based on defined disease names among men.

	Fertility treatment recipients (*n* = 850)		Ages 18–24 (*n* = 5)		Ages 25–29 (*n* = 83)		Ages 30–34 (*n* = 262)		Ages 35–39 (*n* = 270)		Ages 40–44 (*n* = 150)		Ages 45–49 (*n* = 62)		Ages 50–69 (*n* = 18)	
	*n*	%	*n*	%	*n*	%	*n*	%	*n*	%	*n*	%	*n*	%	*n*	%
Defined disease names																
Male infertility	570	67	4	80	60	72	182	69	175	65	97	65	39	63	13	72
Oligospermia	372	44	1	20	41	49	119	45	116	43	56	37	32	52	7	39
Infertility	71	8	1	20	6	7	17	6	30	11	13	9	3	5	1	6
Functional infertility	96	11	1	20	4	5	19	7	41	15	25	17	5	8	1	6
Functional male infertility	24	3	0	0	1	1	5	2	14	5	2	1	2	3	0	0
Primary male infertility	29	3	0	0	3	4	11	4	5	2	6	4	2	3	2	11
Azoospermia	29	3	1	20	8	10	10	4	5	2	5	3	0	0	0	0
Secondary infertility	20	2	0	0	1	1	2	1	10	4	4	3	3	5	0	0
Primary infertility	10	1	0	0	1	1	5	2	2	1	2	1	0	0	0	0
Ejaculatory dysfunction	6	1	0	0	1	1	3	1	0	0	1	1	0	0	1	6
Primary azoospermia	0	0	0	0	0	0	0	0	0	0	0	0	0	0	0	0
Obstructive azoospermia	1	0	0	0	1	1	0	0	0	0	0	0	0	0	0	0
Non‐obstructive azoospermia	0	0	0	0	0	0	0	0	0	0	0	0	0	0	0	0
Inability to ejaculate	0	0	0	0	0	0	0	0	0	0	0	0	0	0	0	0
Unencoded infertility diagnoses^a^	38	4	0	0	4	5	11	4	10	4	11	7	2	3	0	0

^a^ Infertility requiring general infertility treatment or assisted reproductive technology, asthenozoospermia, induction of spermatogenesis in oligospermia, etc.

Tables 5 and 6 show claims for infertility‐specific treatments services and prescriptions for each drug among patients receiving infertility‐specific treatments. Claims for general fertility treatment management fees were highest among women aged 30–34, while claims for ART management fees were highest among women aged 35–39 and 40–44. No claims for ART management fees were observed among women aged 45 and over. Claims for artificial insemination were observed in women aged 45–49, while no claims other than general fertility treatment management fees were observed in women aged 50 and over. Regarding prescriptions, the majority consisted of hormone preparations such as cycle adjustment for ART, luteal supplementation for ART, miscarriage, or premature birth due to luteal insufficiency. Among men, the main type of claim was for general fertility treatment management fees or ART management fees. Less than 5% underwent TESE during the observation period. Among prescriptions, prescription rates were low for all drugs, whereas rates for covered herbal medicines and vitamin supplements were relatively high.

**TABLE 5:** Claims and prescriptions for each drug among female patients receiving infertility‐specific treatments.

	Infertility‐specific treatments recipients (*n* = 3392)		Ages 18–24 (*n* = 15)		Ages 25–29 (*n* = 386)		Ages 30–34 (*n* = 1091)		Ages 35–39 (*n* = 1203)		Ages 40–44 (*n* = 659)		Ages 45–49 (*n* = 37)		Ages 50–69 (*n* = 1)	
	*n*	%	*n*	%	*n*	%	*n*	%	*n*	%	*n*	%	*n*	%	*n*	%
Newly covered fertility treatment services																
General fertility treatment management fee	1873	55	8	53	305	79	717	66	588	49	221	34	33	89	1	100
Assisted reproductive technology management fee	1807	53	3	20	112	29	469	43	735	61	488	74	0	0	0	0
Artificial insemination	1208	36	2	13	162	42	430	39	408	34	181	27	25	68	0	0
Anti‐Müllerian hormone measurement	859	25	4	27	71	18	259	24	331	28	192	29	2	5	0	0
Oocyte retrieval and surcharge	1266	37	4	27	92	24	315	29	494	41	361	55	0	0	0	0
In vitro fertilization management fee	487	14	1	7	28	7	125	11	195	16	138	21	0	0	0	0
Intracytoplasmic sperm injection management fee	647	19	2	13	37	10	133	12	252	21	223	34	0	0	0	0
Simultaneous in vitro fertilization and intracytoplasmic sperm injection management fee	239	7	1	7	30	8	70	6	99	8	39	6	0	0	0	0
Artificial oocyte activation surcharge	71	2	0	0	4	1	11	1	31	3	25	4	0	0	0	0
Zygote/embryo culture management fee	1206	36	4	27	89	23	300	27	475	39	338	51	0	0	0	0
Blastocyst creation surcharge	1110	33	4	27	88	23	288	26	438	36	292	44	0	0	0	0
Embryo cryopreservation management fee (implementation)	1073	32	4	27	87	23	277	25	437	36	268	41	0	0	0	0
Embryo cryopreservation management fee (maintenance)	162	5	0	0	3	1	50	5	71	6	38	6	0	0	0	0
Fresh embryo transfer	200	6	0	0	5	1	39	4	88	7	68	10	0	0	0	0
Frozen embryo transfer	1320	39	4	27	84	22	343	31	558	46	331	50	0	0	0	0
Assisted Hatching surcharge	1041	31	4	27	57	15	250	23	448	37	282	43	0	0	0	0
High concentrations of hyaluronan in culture medium surcharge	821	24	1	7	43	11	205	19	345	29	227	34	0	0	0	0
Drug prescription																
Aromatase inhibitors (letrozole)	849	25	5	33	120	31	305	28	265	22	148	22	6	16	0	0
Clomiphene citrate	1443	43	7	47	205	53	477	44	457	38	277	42	19	51	1	100
Gonadotropin preparations (HMG, FSH, rFSH preparations)	1516	45	5	33	148	38	426	39	554	46	369	56	13	35	1	100
E + P, progestin preparations^a^	2341	69	7	47	278	72	723	66	815	68	489	74	28	76	1	100
Progesterone preparations (luteal phase support)^b^	2402	71	7	47	251	65	716	66	892	74	514	78	22	59	0	0
Progestin preparations (ovulation induction)^c^	1272	38	4	27	158	41	394	36	448	37	256	39	12	32	0	0
Estrogen preparations	1284	38	4	27	88	23	329	30	542	45	320	49	1	3	0	0
GnRH agonists/GnRH antagonists/Human chorionic gonadotropin/Choriogonadotropin alpha	2038	60	5	33	218	56	616	56	730	61	453	69	16	43	0	0

^a^ Those applicable to cycle adjustment in assisted reproductive technology.

^b^ Those applicable to luteal supplementation in assisted reproductive technology or to miscarriage or premature birth due to luteal insufficiency.

^c^ Those applicable to progestin‐primed ovarian stimulation in assisted reproductive technology.

**TABLE 6:** Claims and prescriptions for each drug among male patients receiving infertility‐specific treatments.

	Infertility‐specific treatments recipients (*n* = 519)		Ages 18–24 (*n* = 2)		Ages 25–29 (*n* = 45)		Ages 30–34 (*n* = 158)		Ages 35–39 (*n* = 180)		Ages 40–44 (*n* = 90)		Ages 45–49 (*n* = 35)		Ages 50–69 (*n* = 9)	
	*n*	%	*n*	%	*n*	%	*n*	%	*n*	%	*n*	%	*n*	%	*n*	%
Newly covered fertility treatment services																
General fertility treatment management fee	295	57	0	0	33	73	105	66	86	48	46	51	20	57	5	56
Assisted reproductive technology management fee	253	49	2	100	13	29	64	41	104	58	48	53	17	49	5	56
Y chromosome microdeletion test	12	2	0	0	2	4	4	3	4	2	2	2	0	0	0	0
Conventional testicular sperm extraction	4	1	0	0	2	4	1	1	1	1	0	0	0	0	0	0
Microscopic testicular sperm extraction	12	2	0	0	2	4	6	4	2	1	2	2	0	0	0	0
Drug prescription																
HCG preparations	1	0	0	0	0	0	1	1	0	0	0	0	0	0	0	0
rFSH preparations	3	1	0	0	1	2	2	1	0	0	0	0	0	0	0	0
Clomiphene	9	2	0	0	0	0	3	2	5	3	0	0	1	3	0	0
Sildenafil/Tadalafil	19	4	0	0	0	0	2	1	4	2	9	10	2	6	2	22
Keishi‐bukuryo‐gan (herbal medicine)	5	1	0	0	2	4	1	1	1	1	1	1	0	0	0	0
Hachimi‐jio‐gan (herbal medicine)	3	1	0	0	1	2	1	1	0	0	1	1	0	0	0	0
Hochu‐ekki‐to (herbal medicine)	69	13	0	0	10	22	21	13	17	9	11	12	5	14	5	56
Vitamin B12/Vitamin E	46	9	0	0	11	24	10	6	15	8	7	8	1	3	2	22

**FIGURE 1:**
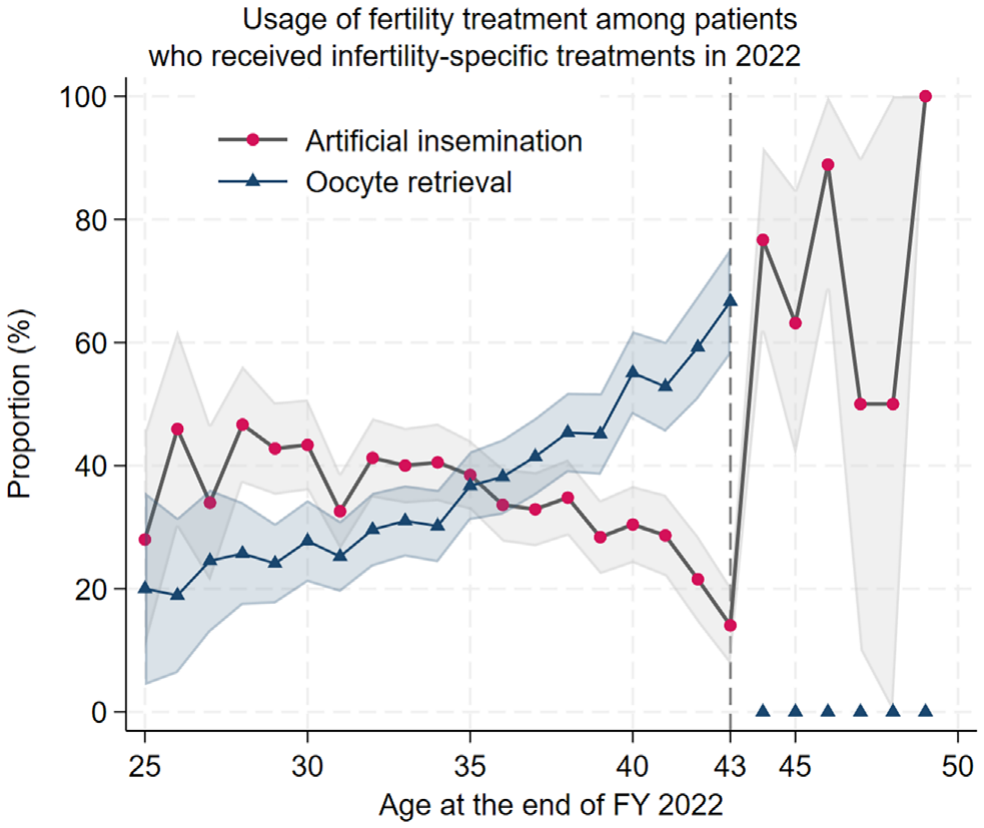
Age‐specific utilization of artificial insemination and oocyte retrieval with 95% confidence intervals. Proportions were calculated as the number of individuals receiving artificial insemination or oocyte retrieval divided by the total number of infertility‐specific treatments recipients within each age. The solid lines represent the age‐specific proportions, and the shaded areas indicate the corresponding 95% confidence intervals. Age was defined at the end of the fiscal year. A vertical dashed line at age 43 indicates the upper age limit at the end of the fiscal year for undergoing oocyte retrieval during the first year of insurance coverage.

Figure 1 shows age‐specific differences were observed in the utilization of artificial insemination and oocyte retrieval. Oocyte retrieval showed a gradual increase with advancing age up to 42 years, whereas artificial insemination demonstrated a peak in the late 20s followed by a decline thereafter. A Mann–Whitney *U* test indicated a significant difference in age distribution between the two procedures (*z* = −9.16, *p* < 0.001), which was consistent with distributional differences confirmed by the Kolmogorov–Smirnov test (*p* < 0.001). When age was categorized, a chi‐square test also demonstrated significant group differences across age strata (*p* < 0.001). These findings indicate that artificial insemination was more common in younger age groups, whereas oocyte retrieval was predominantly chosen among women in their late 30s to early 40s.

Table 7 shows the prevalence of diagnoses of diseases potentially related to fertility treatment among women receiving treatment for infertility, women receiving infertility‐specific treatments and women without a diagnosis of infertility, categorized by age group. While all of these conditions are rare among women without an infertility diagnosis, OHSS, for example, was diagnosed in 8.0% of all women receiving fertility treatment and in 9.6% of patients receiving infertility‐specific treatments. Further, PID, which is potentially associated with oocyte retrieval or artificial insemination, was diagnosed in 1.2% of all women receiving fertility treatment and in 1.3% of patients receiving infertility‐specific treatments.

**TABLE 7:** Prevalence of diagnoses of diseases potentially related to fertility treatment among women.

Age class	Number of each group	Ovarian hyperstimulation syndrome		Bleeding in the ovaries or abdominal cavity		Salpingitis		PID after oocyte retrieval		PID after artificial insemination		PID potentially associated with oocyte retrieval or artificial insemination		Torsion of ovary, ovarian pedicle and fallopian tube		Embolism and thrombosis of vein	
	*n*	*n*	%	*n*	%	*n*	%	*n*	%	*n*	%	*n*	%	*n*	%	*n*	%
Overall																	
Infertility‐specific treatments recipients	3392	325	9.6	17	0.5	33	1.0	3	0.1	2	0.1	45	1.3	1	0.0	48	1.4
Fertility treatment recipients	5167	415	8.0	24	0.5	40	0.8	4	0.1	2	0.0	61	1.2	1	0.0	73	1.4
Not infertility patient	583,153	7	0.0	188	0.0	29	0.0	0	0.0	0	0.0	776	0.1	5	0.0	1694	0.3
20–24																	
Infertility‐specific treatments recipients	15	2	13.3	0	0.0	0	0.0	0	0.0	0	0.0	0	0.0	0	0.0	0	0.0
Fertility treatment recipients	71	2	2.8	0	0.0	1	1.4	0	0.0	0	0.0	1	1.4	0	0.0	0	0.0
Not infertility patient	71,410	0	0.0	41	0.1	3	0.0	0	0.0	0	0.0	87	0.1	0	0.0	86	0.1
25–29																	
Infertility‐specific treatments recipients	386	48	12.4	0	0.0	4	1.0	0	0.0	0	0.0	9	2.3	0	0.0	6	1.6
Fertility treatment recipients	651	54	8.3	1	0.2	6	0.9	0	0.0	0	0.0	10	1.5	0	0.0	7	1.1
Not infertility patient	37,940	2	0.0	29	0.1	4	0.0	0	0.0	0	0.0	59	0.2	0	0.0	97	0.3
30–34																	
Infertility‐specific treatments recipients	1091	100	9.2	5	0.5	12	1.1	1	0.1	0	0.0	13	1.2	0	0.0	17	1.6
Fertility treatment recipients	1630	135	8.3	5	0.3	16	1.0	1	0.1	0	0.0	19	1.2	0	0.0	28	1.7
Not infertility patient	40,216	3	0.0	27	0.1	4	0.0	0	0.0	0	0.0	64	0.2	1	0.0	107	0.3
35–39																	
Infertility‐specific treatments recipients	1,203	127	10.6	8	0.7	8	0.7	1	0.1	1	0.1	16	1.3	1	0.1	19	1.6
Fertility treatment recipients	1721	161	9.4	10	0.6	9	0.5	2	0.1	1	0.1	20	1.2	1	0.1	26	1.5
Not infertility patient	50,116	2	0.0	28	0.1	1	0.0	0	0.0	0	0.0	73	0.1	3	0.0	109	0.2
40–44																	
Infertility‐specific treatments recipients	659	48	7.3	4	0.6	5	0.8	1	0.2	1	0.2	6	0.9	0	0.0	6	0.9
Fertility treatment recipients	970	63	6.5	7	0.7	6	0.6	1	0.1	1	0.1	9	0.9	0	0.0	12	1.2
Not infertility patient	60,759	0	0.0	31	0.1	5	0.0	0	0.0	0	0.0	90	0.1	0	0.0	130	0.2
45–49																	
Infertility‐specific treatments recipients	37	0	0.0	0	0.0	2	5.4	0	0.0	0	0.0	1	2.7	0	0.0	0	0.0
Fertility treatment recipients	116	0	0.0	1	0.9	2	1.7	0	0.0	0	0.0	2	1.7	0	0.0	0	0.0
Not infertility patient	78,043	0	0.0	16	0.0	5	0.0	0	0.0	0	0.0	120	0.2	1	0.0	202	0.3
50–69																	
Infertility‐specific treatments recipients	1	0	0.0	0	0.0	0	0.0	0	0.0	0	0.0	0	0.0	0	0.0	0	0.0
Fertility treatment recipients	8	0	0.0	0	0.0	0	0.0	0	0.0	0	0.0	0	0.0	0	0.0	0	0.0
Not infertility patient	244,669	0	0.0	16	0.0	7	0.0	0	0.0	0	0.0	283	0.1	0	0.0	963	0.4

Abbreviation: PID, pelvic inflammatory disease.

**FIGURE 2:**
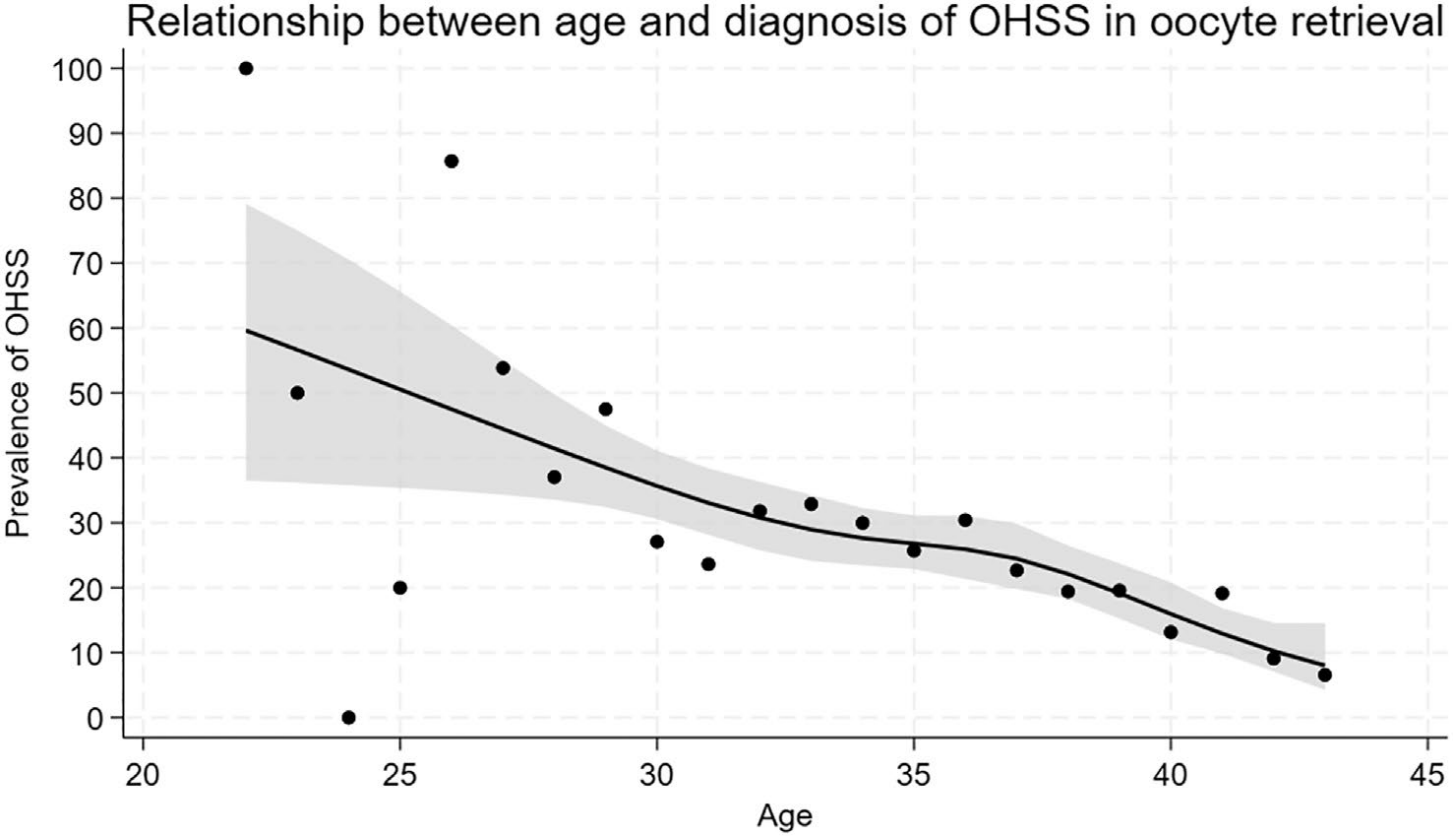
Relationship between age and OHSS diagnosis among patients who underwent oocyte retrieval. Age‐specific predicted probabilities of OHSS with 95% confidence intervals were estimated from a logistic regression model in which age was modeled as a restricted cubic spline with five knots.

Figure 2 and Table S4 show the prevalence of OHSS diagnoses across treatment subgroups and its age‐specific relationship. OHSS was extremely rare among women who did not receive any fertility treatment. In the group with an infertility diagnosis and prescriptions for fertility medications, but without billing for ART or general fertility treatment management fees, the proportion with OHSS peaked at 6.3%, mainly among women in their 30s. OHSS diagnoses were uncommon in the group billed for general infertility treatment management fees but not undergoing ART. Among the 1266 patients who underwent oocyte retrieval, the proportion with OHSS was 12.5% in the 40–44‐year age group and increased towards younger age groups, with approximately half of patients in their 20s receiving an OHSS diagnosis.

Table S5 summarizes the relationships between AMH measurement, ART, oocyte retrieval, and prescriptions of gonadotropin preparations. Among the 859 women who underwent AMH measurement, 729 (84.9%) received ART and 635 (73.9%) were prescribed gonadotropin preparations. By age group, the corresponding proportions were low, at around 25% in the 20–24‐year group, but exceeded 60% in the 25–29‐year group and then gradually increased with advancing age. Across most age groups, approximately half of the women who underwent oocyte retrieval also had AMH measured, with no clear age‐related trend observed.

## 4 Discussion

This study of the prevalence of fertility treatments covered by public health insurance since April 2022, based on disease names and medical claims, revealed a high demand for fertility treatment among women. The highest percentage of fertility treatment patients was among women aged 30 to 34 years. Regarding the infertility‐specific treatments specific to fertility issues, one in 25 women in this age group received treatment. In contrast, for fertility treatment involving the combination of a diagnosis and prescription, the ratio was one in 20. Of note, these data included unmarried women and those who did not wish to have children, and thus the percentage of women who wished to have children and received fertility treatment is likely to have been even higher.

In treatment choices under insurance coverage, the utilization of artificial insemination decreased with increasing age, while that of oocyte retrieval increased with advancing age, with the utilization reversing at age 35. Since oocyte retrieval has an upper age limit of 42, individuals turning 44 by the end of the fiscal year were ineligible. Consequently, the proportion of oocyte retrieval drops to zero at age 43. Therefore, for those receiving infertility‐specific treatments at age 44 and older, the proportion of artificial insemination claims shows a clear increase, diverging from the previous downward trend. This indicates a persistent desire for childbearing even beyond the age restrictions for ART under insurance claims. According to Japan's 2022 ART outcome report, pregnancy rates for women aged 43 and older are below 20%, with a miscarriage rate of 47%. There is a need to utilize new technologies such as oocyte cryopreservation and to discuss future pregnancy and fertility treatment plans at an earlier stage [9].

In contrast to the high prevalence of fertility treatment among women, it was low among men. Even in the 30–34 age group, which had the highest prevalence, only 0.3% of men had infertility‐specific treatments and 0.5% received fertility treatment. We suggest three possible reasons for this. First, men may be reluctant to visit an infertility clinic or undergo treatment. Previous studies have shown that many male patients only visit a clinic at their spouse's request, and that there are psychological barriers to diagnosis and treatment, with few cases of voluntary testing [10]. Indeed, even among couples who have undergone fertility treatment, only 11% of men have received treatment [6]. Second, few treatment options are likely available to men. With artificial insemination and ART, if motile sperm can be selected from sperm obtained by sperm collection, no further treatment is required. It is therefore possible that no diagnosis or medical claim is made because no active treatment is performed. Third, few facilities perform TESE: as of July 2022, while 610 facilities were able to bill for ART management fees, only 174 could bill for TESE [11]. Barriers to accessing medical care for male infertility may contribute to the low prevalence of treatment for this condition.

The prevalence of azoospermia is estimated at 1% of all men and 10%–15% of infertile men [12, 13]. Generally, conventional TESE or vasectomy reversal is performed for obstructive azoospermia, while micro‐TESE is an important option for non‐obstructive azoospermia [14, 15, 16]. Among the whole 619,551 men in this study, only 54 (0.01%) had azoospermia registered as a diagnosis, and only 29 (3.4%) were among those receiving fertility treatment. However, when limited to fertility treatment recipients aged 25–29, 8 (10%) were diagnosed, consistent with previous reports [12]. The gap between previously reported epidemiological findings and the diagnosis ratio in this study stems from the fact that individuals without a desire for pregnancy and who do not require fertility treatment have no opportunity to have the diagnosis registered at a medical institution. Therefore, the proportion in this study should be interpreted as the proportion of diagnosed azoospermia cases, not the actual prevalence of azoospermia. Among the 29 azoospermia patients undergoing some form of fertility treatment, 16 (approximately half) utilized TESE. The breakdown was 4 cases of conventional TESE and 12 cases of micro‐TESE. This dataset did not observe any diagnosis registrations specifically for non‐obstructive azoospermia; in most cases, the diagnosis did not distinguish between non‐obstructive and obstructive types. Consequently, this study could not determine which treatment options were selected for which specific types of azoospermia.

This study examined diagnoses commonly given to infertile patients. Our ability to capture the majority of fertility treatment recipients using predefined diagnoses demonstrates the effectiveness of standardizing diagnostic codes following the introduction of health insurance coverage: specifically, before coverage, attempts to understand the actual status of diagnoses and treatments nationwide were hampered by diagnosis‐dependent differences in examinations and treatment content among hospitals. In fact, a nationwide survey conducted by Shionoya et al. immediately before the introduction of health insurance coverage revealed significant differences in the adoption rate of additional in vitro fertilization treatment methods between facilities. Outpatient clinics showed a notably higher adoption rate than hospitals and university hospital [17]. The availability of unified diagnosis codes and information on the status of medical claims (e.g., management fees, treatments, and medical examinations) is expected to further improve the comprehensiveness and accuracy of disease classification and treatment.

This study also revealed the specifics of treatments and prescribed drugs. There are various methods of administering fertility treatment drugs and policies. In their 10‐year international survey, for example, Shoham et al. reported the lack of a single accepted protocol even for luteal phase replacement therapy [18]. Our present study demonstrates the potential of collecting data from facilities using standardized diagnostic and medical procedure codes for fertility treatment. In particular, collection will enable the analysis of treatment outcomes, which will in turn allow an objective understanding of the current state of medical care and facilitate improved treatment protocols.

The observed OHSS diagnosis proportion among patients who received fertility treatment in this study (8.0%–9.6%) was markedly higher than the OHSS incidence rate (0.79%) reported in previous clinical studies [19]. There are several possible reasons for this discrepancy. First, a diagnosis of OHSS is required for health insurance coverage of cabergoline prescription, a diagnosis may be made in advance when cabergoline is administered prophylactically in cases with a high risk of OHSS. Second, the reported clinical studies were limited to moderate to severe OHSS, whereas the claims data may include mild and suspected cases. OHSS is a representative complication of oocyte retrieval and controlled ovarian stimulation [20]. Elevated AMH levels are a risk factor for OHSS, and for follitropin delta, the dosage is adjusted based on AMH measurement results [21, 22]. As observed in this study, in younger age groups, responsiveness to gonadotropin preparations is generally higher, resulting in more follicles and a higher risk of developing OHSS [23]. The pattern of AMH measurement observed in this study was closely linked to decisions regarding oocyte retrieval and controlled ovarian stimulation. Of the 1266 patients who underwent oocyte retrieval, approximately half had undergone AMH measurement. Although the restriction of AMH testing being limited to once every 6 months may underestimate its use prior to insurance coverage, half of the cases underwent oocyte retrieval without AMH measurement. AMH measurement is crucial for assessing step up to ART and evaluating OHSS risk. AMH measurement should be performed in more cases, and in younger patients or those with extremely high AMH levels (e.g., due to PCOS), more cautious ovarian stimulation tailored to the AMH value is necessary [24, 25].

This study has a number of limitations inherent to observational studies that use medical claims data. The disease names in this study are those registered by medical institutions and may differ from actual diagnoses. They may include so‐called “insurance disease names” added to ensure consistency with prescriptions or treatments, as mentioned above with regard to cabergoline prescriptions. Studies using medical claim data sometimes compare data with actual medical records to verify the validity of diagnoses. However, since fertility treatment is performed by various general practitioners in different locations, we determined that comparing data with medical records from specific institutions alone would not be sufficient to verify the validity of diagnoses. Therefore, in this study, we aimed to accurately capture these diagnoses by combining them with prescriptions and infertility‐specific treatments. Rare diseases and complex conditions not included among the diagnoses defined in this study require further investigation. Additionally, this study used data from the first year that fertility treatment was covered by health insurance. The system may not have been fully covered, and the provision of treatment and visits to clinics covered by insurance may have been underestimated. Additionally, for men, medical procedures defining fertility treatment, such as TESE, are often not performed cyclically and repeatedly, unlike for women. Therefore, if medical procedures were already being performed before coverage began, data from the first year alone would underestimate the actual volume. Future research should employ data spanning multiple years to conduct longitudinal analyses. Finally, the member companies enrolled in this health insurance database were relatively large. The socioeconomic status of the covered population was therefore relatively high and these employees and their dependents may have had easier access to medical care than the general population and more full‐time workers may delay marriage and pregnancy, increasing the likelihood of needing fertility treatment. This could lead to an overestimation of the number of patients. On the other hand, higher socioeconomic status and more full‐time workers may also mean more unmarried individuals or couples who do not desire children, potentially lacking the time for fertility treatment, which could lead to an underestimation. The total direction of these biases is uncertain, and future research is needed to compare the results with those from the National Database of Health Insurance Claims or other datasets for unique individuals.

## 5 Conclusions

This study described the actual state of clinical practice in Japan during the first year of health insurance coverage for fertility treatment. Between 2.6% and 3.9% of women aged 30–34 years, a population which includes unmarried women and those who did not wish to have children, had received some form of fertility treatment. Fewer men received fertility treatment than women, indicating the need to raise awareness of the availability of medical consultations, examinations, and treatment, improve access to medical care, and expand treatment options.

## Funding

This work was supported by Japan Society for the Promotion of Science, JP24K20229.

## Conflicts of Interest

Takeshi Iwasa is an Editorial Board member of Reproductive Medicine and Biology and a co‐author of this article. To minimize bias, they were excluded from all editorial decision‐making related to the acceptance of this article for publication.

## Supporting information


**Table S1:** Definitions of infertility diagnoses and fertility treatment for men and women.

**Table S2:** Coverage ratios based on defined disease names and representative disease names observed but not included in the definitions in women.

**Table S3:** Coverage ratios based on defined disease names and representative disease names observed but not included in the definitions in men.

**Table S4:** OHSS diagnosis ratios at each subgroup by treatment type.

**Table S5:** The association between AMH measurement and ART, oocyte retrieval, and prescriptions.

## Data Availability

The data that support the findings of this study are available on request from the corresponding author. The data are not publicly available due to privacy or ethical restrictions.
